# Maternal nutrition at conception modulates DNA methylation of human metastable epialleles

**DOI:** 10.1038/ncomms4746

**Published:** 2014-04-29

**Authors:** Paula Dominguez-Salas, Sophie E. Moore, Maria S. Baker, Andrew W. Bergen, Sharon E. Cox, Roger A. Dyer, Anthony J. Fulford, Yongtao Guan, Eleonora Laritsky, Matt J. Silver, Gary E. Swan, Steven H. Zeisel, Sheila M. Innis, Robert A. Waterland, Andrew M. Prentice, Branwen J. Hennig

**Affiliations:** 1 MRC International Nutrition Group at MRC Keneba, The Gambia and London School of Hygiene & Tropical Medicine, EPH, LSHTM, Keppel Street, London WC1E 7HT, UK; 2 Department of Pediatrics, Baylor College of Medicine, USDA/ARS Children's Nutrition Research Center, 1100 Bates Street, Houston, Texas 77030, USA; 3 Center for Health Sciences, SRI International, 333 Ravenswood Avenue, Menlo Park, California 94025-3493, USA; 4 Department of Pediatrics, University of British Columbia, Room 179, Child and Family Institute, 950 West 28th Avenue Vancouver, British Columbia, Canada V5Z 4H4; 5 Department of Molecular & Human Genetics, Baylor College of Medicine, Houston, Texas 77030, USA; 6 Stanford Prevention Research Center, Stanford University School of Medicine, Stanford, California 94305-5411, USA; 7 Nutrition Research Institute, Department of Nutrition, Gillings School of Global Public Health and School of Medicine, University of North Carolina at Chapel Hill, Chapel Hill, 500 Laureate Way, Kannapolis, North Carolina 28081, USA

## Abstract

In experimental animals, maternal diet during the periconceptional period influences the establishment of DNA methylation at metastable epialleles in the offspring, with permanent phenotypic consequences. Pronounced naturally occurring seasonal differences in the diet of rural Gambian women allowed us to test this in humans. We show that significant seasonal variations in methyl-donor nutrient intake of mothers around the time of conception influence 13 relevant plasma biomarkers. The level of several of these maternal biomarkers predicts increased/decreased methylation at metastable epialleles in DNA extracted from lymphocytes and hair follicles in infants postnatally. Our results demonstrate that maternal nutritional status during early pregnancy causes persistent and systemic epigenetic changes at human metastable epialleles.

## Abstract (web summary)

Maternal diet affects DNA methylation in the developing offspring, leading to phenotypic changes. Here, Dominguez-Salas *et al*. exploit seasonal variation in the diet of Gambian women to show that maternal methyl donor nutrient status around the time of conception predicts methylation levels at metastable epialleles in infants.

## Graphical abstract

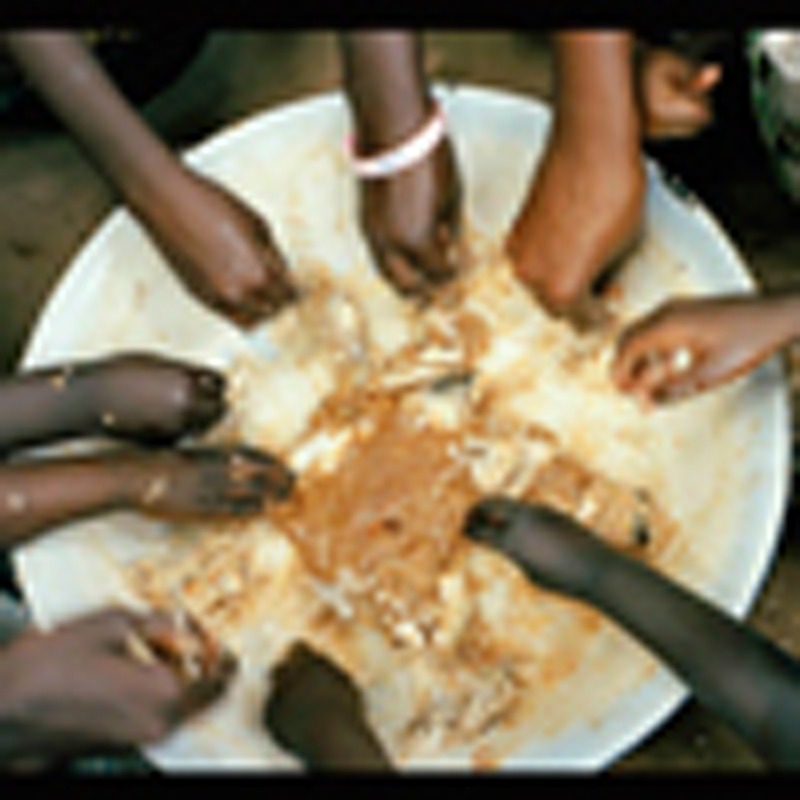

Metastable epialleles (MEs) are genomic regions at which DNA methylation is established stochastically in the early embryo then stably maintained in differentiated tissues, leading to interindividual epigenetic variation that affects multiple cell types^1^. Experiments in *agouti viable yellow* (*A*^*vy*^) mice, later confirmed in *axin fused* (*Axin*^*Fu*^) mice, demonstrated that maternal dietary changes affecting methyl-donor availability alter epigenetic development at MEs, causing permanent phenotypic variation among isogenic offspring^2^,^3^,^4^. MEs are essentially epigenetic polymorphisms; their methylation in accessible cells (for example, peripheral blood lymphocytes (PBL)) provides a readout of the epigenetic state in other tissues, making them attractive candidates for epigenetic epidemiological studies^5^,^6^. Exploiting an experiment of nature that determines seasonal fluctuations in the dietary intake and nutritional status of rural Gambian women, we previously demonstrated that season of conception significantly influences the methylation of candidate MEs in children^7^. Contrary to our initial hypothesis, the percentage of DNA methylation was higher in children conceived in the protein-energy-limited rainy (‘hungry’) season than in those conceived in the dry (‘harvest’) season. We therefore hypothesized that rather than the negative energy balance observed in mothers during the rainy season, other nutrients critical to methyl-donor metabolic pathways may have a limiting role^7^.

We here report a prospective study that replicates the season of conception effects on epigenotype and extends the findings to additional human MEs. We further show that DNA methylation is predictably influenced by periconceptional maternal plasma biomarker concentrations of key micronutrients involved in one-carbon metabolism. This represents a demonstration in humans that a mother’s nutritional status at the time of conception can influence her child’s epigenome, with likely lifelong implications.

## Results

### Seasonal differences in maternal blood biomarkers

In rural Gambia the combination of a monomodal rainy season and the population’s primary dependence on the consumption of own-grown foods leads to profound annual variations in the intakes of macro and micronutrients. Such variations are compounded by the seasonally variable agricultural workload^8^. These annual oscillations in nutrient availability and substrate utilization have long been known to affect foetal growth and development^9^,^10^,^11^, offering a powerful means to explore mechanisms by which early diet affects long-term functional outcomes in humans. To exploit this model we designed a prospective study to map the influence of mothers’ periconceptional dietary intakes and plasma concentrations of key methyl-donor pathway substrates (methionine (MET), choline (CHOL), betaine (BET)) cofactors (folate (FOL), vitamins B2, B6, B12, active B12 (ACTB12)) and intermediary metabolites (dimethyl glycine (DMG), S-adenosylmethionine (SAM), S-adenosylhomocysteine (SAH), homocysteine (HCY), cysteine (CYS)) on their infants’ DNA CpG methylation at seven curated MEs. Because it would be impractical to collect repeated dietary intake and blood draws in hundreds of (pre-)pregnant women to capture their precise metabolic status at the time of conception, we implemented a two-part design using separate groups of women living among the same villages. An ‘indicator’ group of non-pregnant women (*N*=30) had dietary intake and plasma biomarker concentrations assessed monthly over a full calendar year (July 2009 to June 2010) to provide data to model the seasonality of each variable using truncated-Fourier series^12^. These results have been published in full elsewhere^13^. Concurrently, another group of women (the ‘main’ group, *N*=2,040) were followed while non-pregnant and visited monthly until a missed menses was reported, at which point a blood draw was taken for plasma biomarker assessment; pregnancy was confirmed a month later. Women who conceived at the peak of the rainy (‘hungry’) season (July–September 2009, *N*=84) or the peak of the dry (‘harvest’) season (February–April 2010, *N*=83) were fully enroled into the main group (see Methods). The sonographically-determined stage of gestation at the time of sampling was 8.6±4.0 weeks (mean±s.d.). Predicted biomarker concentrations at the time of conception were calculated by back extrapolation (adjusting for season and gestational age) using the modelled data from the indicator group and allowing for pregnancy-induced changes in substrate concentrations (see Methods, Supplementary Tables 1 and 2).

**Figure 1:**
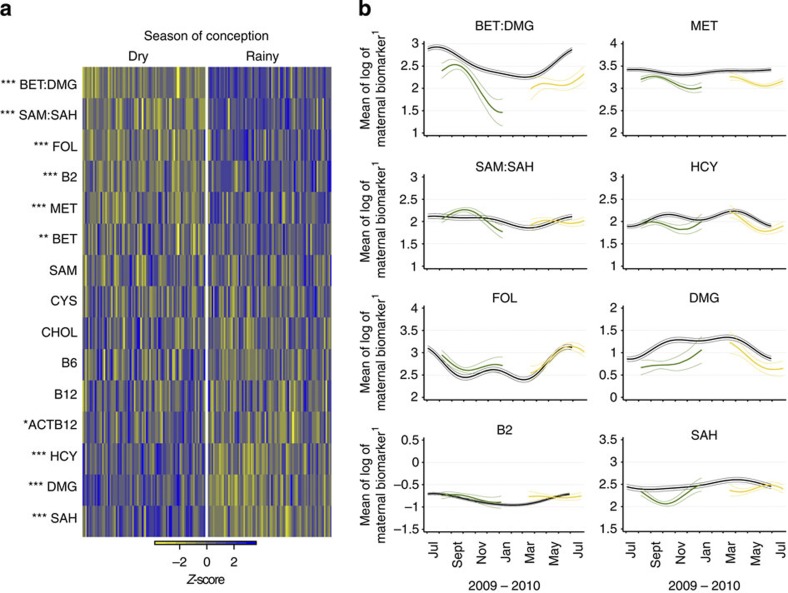
Seasonal differences in plasma biomarker concentrations. (**a**) Heatmap of seasonal variation in maternal plasma biomarker concentrations at the time of conception. Columns correspond to pregnant women (main group), grouped according to season of conception. Colours represent deviation from all season mean biomarker concentrations, calculated as *z*-scores. Biomarkers are ranked by mean seasonal difference (Supplementary Table 2), with the greatest increment in the rainy versus dry season (BET:DMG) at the top, and the greatest decrement (SAH) at the bottom. Analysis of variance *P*-values: *<0.05; **<0.01; ***<0.001; *N*=167. (**b**) Comparison of seasonal patterns in the plasma biomarker concentrations between the indicator group (*N*=30, shown in black) and the main group before back extrapolation (*N*=167 total; rainy season (green), dry season (yellow)) for BET:DMG and SAM:SAH ratios, FOL, B2, MET, HCY, DMG and SAH. Y-axis units: FOL and SAH nmol l^−1^; in MET, HCY and DMG μmol l^−1^; B2 in 1 EGRAC^−1^. Thick line, mean of logarithm of the biomarker; thin lines, 95%CI. Plots for the remaining biomarkers are shown in Supplementary Fig. 1. B2 (riboflavin) is represented as the reciprocal of the erythrocyte glutathione reductase activation coefficient (EGRAC), a functional test inversely associated with red blood cell riboflavin sufficiency.

Eight of the 13 biomarkers, and two derived variables (BET:DMG and SAM:SAH) showed significant differences between seasons of conception (Fig. 1a and Supplementary Table 2). Maternal periconceptional concentrations of FOL, B2, MET, BET and the SAM:SAH and BET:DMG ratios were higher in the rainy season, and concentrations of ACTB12, DMG, HCY and SAH were lower. Biomarker-specific seasonality in the main and indicator groups are illustrated in Fig. 1b and Supplementary Fig. 1.

### Maternal predictors of infant DNA methylation

**Figure 2:**
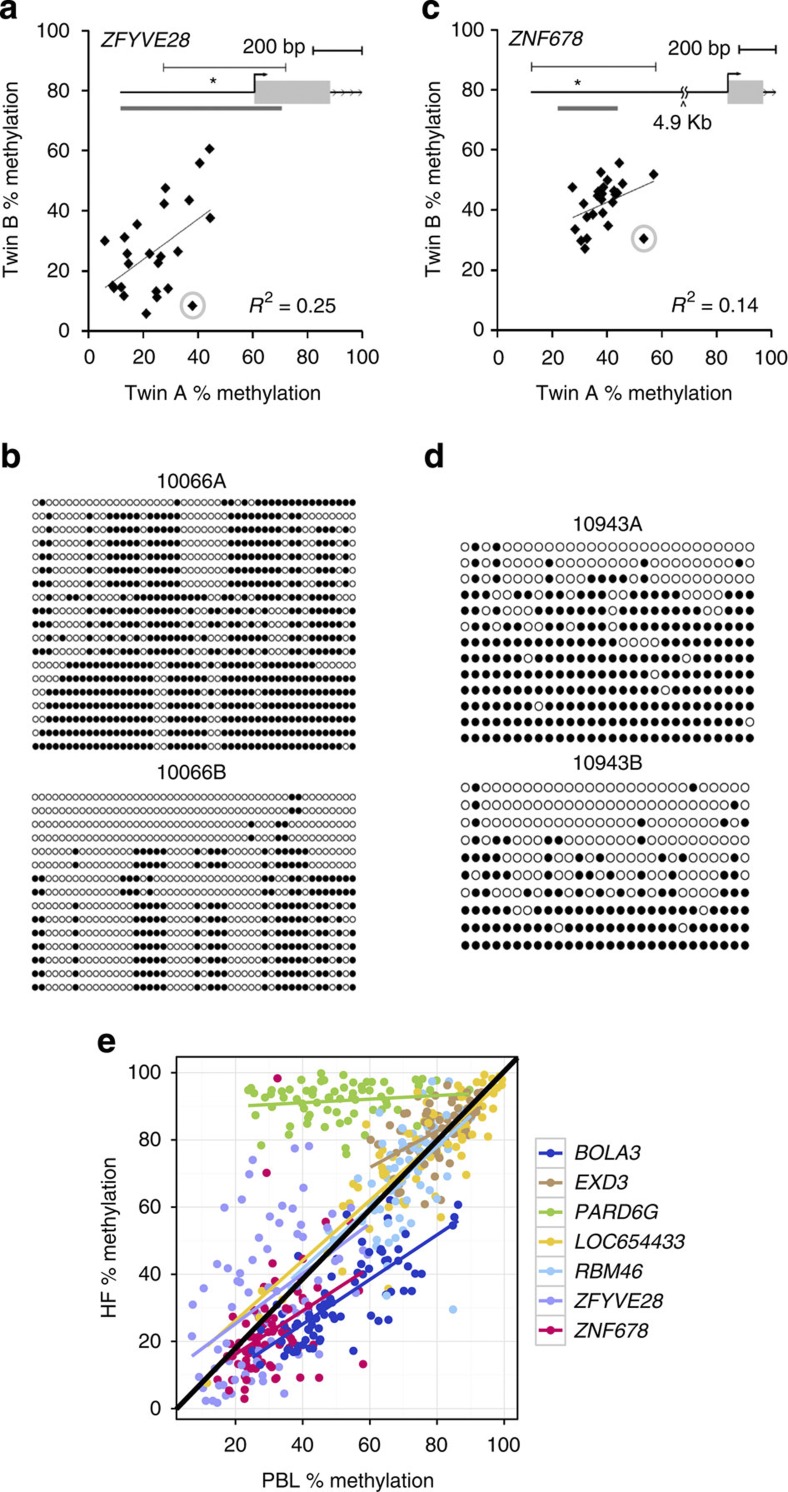
Examples of verification of metastable epialleles in humans. (**a**) Pearson correlation of PBL DNA methylation at *ZFYVE28* within 25 pairs of MZ twins^15^ shows that most interindividual variation in DNA methylation is not explained by genetics. (The inset shows the genomic region; the grey bar below the gene indicates a CpG island, and the asterisk the location of the pyrosequencing assay.) (**b**) Clonal bisulfite sequencing of discordant twin pair 10066 (circled in (**a**)). Each row represents an individual clone from the post-bisulfite PCR product, and each column a CpG site. Filled circles indicate methylation. The ~500 bp region analyzed is indicated by the line above the gene in (**a**). Not only the degree but also the CpG site-specific pattern of methylation is highly discordant between the two isogenic individuals. (**c**) Pearson correlation of PBL DNA methylation for *ZNF678* within 25 MZ twin pairs again illustrates that interindividual variation is not genetically mediated. (**d**) Clonal bisulfite sequencing of discordant twin pair 10943 (circled in (**c**)). (**e**) DNA methylation is highly correlated between PBL and HF across all MEs (excluding *PARD6G*, average Pearson correlation coefficient *r*=0.72, range 0.39 *(ZNF678)* to 0.87 (*LOC654433*)), *N*=82 paired Gambian PBL and HF samples). PBL originate from mesodermal and HF from ectodermal germ layers of the early embryo; thus these data confirm that the systemic interindividual variation demonstrated in Vietnamese adults (Supplementary Fig. 2) generally extends to these Gambian children.

We tested whether these nutritionally driven seasonal differences in maternal periconceptional one-carbon metabolism affect the establishment of DNA methylation at human MEs. The DNA methylation analyses focused on four previously described MEs (*BOLA3*, *LOC654433*, *EXD3* and *ZFYVE28* (ref. 7)), and three additional loci (*RBM46*, *PARD6G* and *ZNF678*). The latter novel MEs were identified by methylation-specific amplification microarray analysis^14^, using a parallel two-tissue co-hybridization design to detect systemic interindividual variation^7^. Validation experiments using autopsy samples from Vietnamese adults demonstrated high inter-tissue correlation of DNA methylation among distinct embryonic germ layer lineages (liver, kidney and brain) (Supplementary Fig. 2), and epigenetic discordance within United States monozygotic (MZ) twin pairs from a previously established twin registry^15^ (Fig. 2a–d) corroborated these loci as true human MEs.

**Figure 3:**
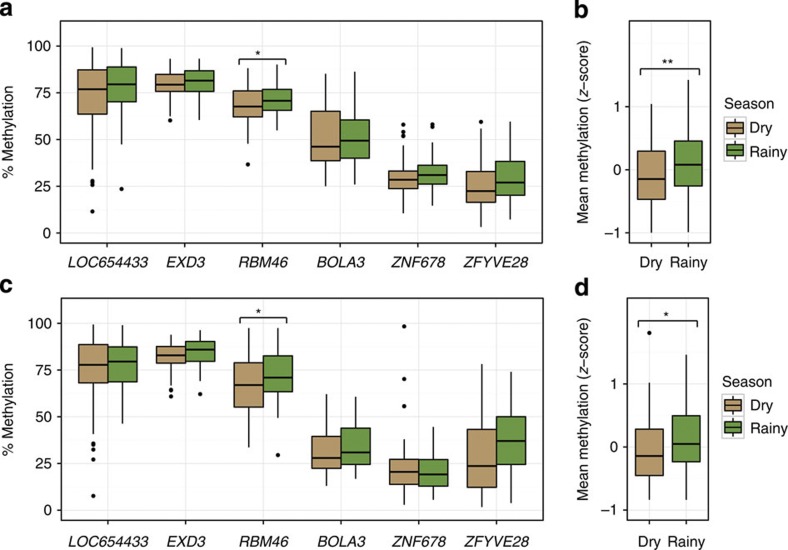
Season of conception affects DNA methylation at MEs. (**a**) Percent methylation at the six MEs in PBL of infants conceived in the dry or rainy season. Median % methylation is consistently higher in infants conceived in the rainy season. (**b**) Mean PBL methylation *z*-score across the six MEs is significantly higher in infants conceived in the rainy season. (**c**) Percent methylation at the six MEs in HF of infants conceived in the dry or rainy season; the overall pattern of methylation is similar to that observed in PBL, as is the seasonal difference in mean methylation *z*-score (**d**). Box plots represent the median (horizontal line) and interquartile range (box) of the indicated distribution. The whiskers extend from the top/bottom of the box to the highest/lowest data value that is within 1.5. Asterix represents interquartile range of the box. Data beyond the whiskers are plotted as individual points. PBL, peripheral blood lymphocyte; Oneway analysis of variance *P*-values: *<0.05, **<0.01; PBL *N*_max_=126 and HF *N*_max_=87 infant DNA samples.

In mice, effects of maternal nutrition on DNA methylation at MEs are established in the early embryo and subsequently propagated to all germ lineages^4^,^16^. To test whether human MEs behave similarly, we obtained PBL (*N*=126) and hair follicle samples (HF, *N*=87) mesodermal and ectodermal tissues, respectively from 2–8 month (3.6±0.9) old infants born to mothers in the main group. Perhaps owing to ‘limited lineage’ epigenetic metastability^3^, *PARD6G*^5^ was consistently hypermethylated in HF (Fig. 2e) and therefore excluded from subsequent analyses. As expected, percent methylation in HF and PBL within each of the remaining six MEs was highly correlated (average *r*=0.72; Fig. 2e). Importantly, effects of season of conception on DNA methylation were also correlated in the two tissues. Offspring of rainy season conceptions had higher levels of CpG methylation at the six remaining MEs in PBL (Fig. 3a) and the overall effect of season was highly significant (Fig. 3b). The seasonal pattern in HF samples was similar (Fig. 3c,d).

We used a 7-step approach to build linear least squares regression models to regress the methylation scores for the individual MEs and the mean methylation scores across all MEs against season, against each maternal nutritional status biomarker and against other covariates (see Methods). There was no evidence for ME-specific effects of any of the biomarkers, the derived variables, or other covariates with the exception of infant sex (seeming unrelated regression (SUR) *P*=0.0003). Therefore the combined sex-adjusted mean methylation score for the six MEs was used in the final analysis. Maternal body mass index (BMI), vitamin B2, HCY and CYS concentrations at the time of conception predicted mean ME methylation in both PBL and HF DNA of their infants (Table 1). Association was also detected for B6 in PBL. Increased maternal periconceptional CYS and HCY concentrations predicted decreased systemic methylation (that is, both tissues) in their infants, whereas maternal B2 concentrations predicted increased ME methylation. The direction of each of these associations is congruent with their expected impact on methyl group supply. The percentage of the variance of methylation of PBLs explained by all the biomarkers combined was 10.3%, 95%CI=(7.7, 38.5%); the ‘true’ association, after adjusting for correlation dilution imposed by estimating the biomarkers in a single blood draw and back extrapolating to conception^13^, is likely much stronger. While biomarkers both vary seasonally and are strongly associated with DNA methylation, their net effect does not fully explain the seasonal variation in DNA methylation; the seasonal effect remained significant with an increased coefficient (−0.61, 95%CI=(−1.11, −0.11); multiple regression model *P*=0.018) when the regression model included all the biomarkers.

## Discussion

Evidence is accumulating that environmental factors during early-life have long-term effects on later health outcomes and that these processes reflect epigenetic responses to periconceptional exposures^17^. Our data represent first-in-human confirmation that the maternal blood biomarker status of substrates and cofactors required for methyl-donor pathways, measured around the time of conception, predicts the methylation patterns of MEs in offspring. Increased maternal BMI was also predictive of decreased systemic infant DNA methylation at MEs. This finding is potentially of global significance and is the subject of further studies in which we are attempting to distinguish possible effects driven by total adiposity and/or dynamic changes in energy balance. Further research beyond the current set of MEs and the follow-up of infants from this study is also ongoing.

Our experimental design (based on random distribution of conceptions to different seasons) eliminates many possible confounders and, because our data corroborate prior knowledge from controlled supplementation studies in animal models^2^,^3^,^4^,^6^, a likely causal effect can be inferred. Although the phenotypic consequences of these variations in methylation are not yet known, the possible implications of tissue-wide epigenetic variation at MEs induced by subtle differences in maternal micronutrient status and BMI at the time of conception are far reaching.

## Methods

All procedures were approved by the joint Gambian Government/MRC Ethics Committee and written-informed consent was obtained from all participants or their guardians.

### Study setting and population

This was an observational prospective cohort study, conducted between July 2009 and July 2011 in 34 villages across the rural West Kiang district of The Gambia, within the catchment area of the MRC International Nutrition Group’s field station at MRC Keneba ( http://www.ing.mrc.ac.uk). This study was registered at http://www.clinicaltrials.gov, reference number NCT01811641, as a proof-of-principle observational study.

*‘Indicator’ group women:* Thirty non-pregnant, non-lactating women from three villages were followed monthly for one full calendar year for the assessment of dietary intake (48 h-weighed records) of nutrients involved in methyl-donor pathways and their effect on respective metabolic plasma biomarker concentrations by season in a parallel study (for full details see ref. 13).

*‘Main’ group mothers:* All women of reproductive age (18–45 years) registered in the MRC ING’s Demographic Surveillance System for West Kiang in The Gambia (DSS, http://www.ing.mrc.ac.uk/research_areas/west_kiang_dss.aspx) were invited to participate; 2040 women consented. Exclusion criteria included confirmed pregnancy at time of recruitment, menopause or likely migration (short or long term) away from West Kiang. Each month all 2040 women were assessed at the village health post for weight (Tanita DH305 scales (Tanita Corporation, Japan) and height measurement (Leicester stadiometer, Seca 214, UK)) and answered a short questionnaire on the date of their last menstrual period. On the first report of a missed menses, a 10 ml fasting venous blood sample was collected for the purpose of plasma biomarker assessment. Upon reporting of a second consecutive missed period the following month, a urine sample was collected for pregnancy testing (this system was set up to avoid early disclosure of pregnancy to which some women objected). If the test was negative the woman continued to be visited monthly, and her blood sample from the previous month was discarded. If the test was positive, the woman was invited to the MRC Keneba field station for confirmation and dating of pregnancy by ultrasound examination and a full antenatal check. Women who conceived during the peak of the rainy (July–September 2009) or dry (February–April 2010) season and with a maternal blood sample collected within the first 16 weeks from conception were then fully enroled. Multiple pregnancies were excluded. The total number of women who conceived during the *a priori* selected months and having a blood sample collected during the first 16 weeks of pregnancy from conception was 166, recruited across 24 villages. By study design, conceptions were randomly allocated to the different seasons and therefore village was not considered as covariate in the analyses.

*‘Main’ group infants:* Between 2–8 month (3.6±0.9) (mean, s.d.) after delivery infant samples of venous blood (3 ml) and HFs were collected by a trained nurse for the purpose of DNA extraction. A total of 126 PBL and 87 HF samples were obtained. Fewer HF samples were collected owing to some mothers objecting as a number of children had too little hair for sampling leading to insufficient DNA harvested from HF.

Summary statistics of the study population are shown in Supplementary Table 1.

### Maternal blood methyl-donor and co-factor concentrations (biomarkers)

The first day of the last menses is estimated to be 14 days before fertilization. Conception date was thus calculated by adding 14 days to the estimated date of onset of a woman’s last menses, based on the gestational age determined by ultrasound at the time of the first antenatal check.

Plasma biomarker measurements included FOL, B2 (by functional test, see below), B6, B12, ACTB12 (holotranscobalamin, the biologically ACTB12), CHOL, BET and MET, as well as HCY, SAM, SAH and DMG. Maternal blood biomarker assessment was carried out using the same methodologies as for the indicator group women, as described previously^13^. Briefly, maternal blood samples (10 ml in EDTA tubes) were collected in the field and transported on ice to the MRC Keneba laboratory for processing and freezing within a maximum of 2 h, to avoid decay of any of the biomarkers (for example, SAM to SAH conversion). Blood samples were spun at 2,750 *g* for 10 min, the plasma taken off and frozen at −80 °C immediately. A sample of red blood cells was removed from the plasma-depleted blood fraction, washed and stored at −80 °C. All plasma biomarkers except B2 were assessed at the Department of Paediatrics, University of British Columbia, Canada. SAM, SAH, CHOL, BET, DMG, HCY, MET, CYS and B6 were analyzed by liquid chromatography–tandem mass spectrometry. B12, ACTB12 and FOL were analyzed by a microparticle enzyme intrinsic factor assay and by ion capture assay respectively, on an AxSyM analyzer (Abbot Laboratories, Chicago, IL, USA). B2 status was determined in red blood cell lysate at MRC Human Nutrition Research (HNR), Cambridge, UK, using the erythrocyte glutathione reductase activation coefficient (EGRAC) assay, performed on a microplate. Higher EGRAC values denote B2 deficiency.

### DNA methylation

Infant PBL DNA was extracted from venous blood using a standard salting-out method^18^ and extracted DNA was cleaned using the Chelex-100 (BIO-RAD) protocol. Infant DNA from HFs was extracted by phenol chloroform extraction and ethanol precipitation, as previously described^7^. DNA methylation analysis was carried out at the Baylor College of Medicine, USDA/ARS Children's Nutrition Research Center, Houston, Texas, USA. Four previously described MEs^7^, namely *BOLA3*, *LOC654433*, *EXD3* and *ZFYVE28*, were assessed in the current study. (An additional locus, *SLITRK1*, was eliminated based on a strict cutoff (*R*^2^>0.50) in the inter-tissue correlation comparison of DNA methylation in the expanded set of Vietnamese adults.) In addition, three newly identified MEs (*RBM46*, *PARD6G* and *ZNF678*) were investigated. These new MEs were determined as previously described employing a custom methylation-specific amplification microarray^14^ combined with a multiple-tissue screening procedure with validation by bisulfite pyrosequencing^3^. CpG site-specific methylation in the current infant DNA samples was measured by quantitative bisulfite pyrosequencing (Pyro Gold reagents and a PSQTM HS 96 pyrosequencer, both from Biotage), as described elsewhere^2^. Briefly, 0.5–2 μg of genomic DNA was bisulfite treated, followed by locus-specific PCR amplification and pyrosequencing to measure methylation at 4 to 12 CpG sites per candidate locus (Supplementary Table 3). Each pyrosequencing assay covered 50–70 bp of DNA and was initially validated by analyzing 0, 25, 50, 75 and 100% methylated human genomic DNA standards^19^ (Supplementary Fig. 4).

The MZ twin peripheral blood DNA samples were drawn from the Northern California Twin Registry^15^; polymorphisms were genotyped to determine zygosity. Post-mortem liver, kidney and brain tissues from Vietnamese motor vehicle accident victims was obtained from a human tissue bank (ILSbio, LLC, Chestertown, MD, USA)^7^.

### Statistical analyses

We set out to test: (i) whether methylation of the seven MEs varied according to the season of conception, (ii) whether plasma biomarkers associated with one-carbon metabolism were predictive of ME methylation; and (iii) whether changes in the availability of methyl donors (as reflected by plasma biomarkers) explain the seasonal difference in ME methylation. The analysis followed seven steps:

1. Back extrapolation of biomarker concentrations: Blood samples for biomarker measurements were collected within 0–16 weeks (mean 8.6±4.0 weeks) post conception. To estimate biomarker concentrations at the time of conception we back-extrapolated along a trajectory parallel to the seasonal patterns (fitted by Fourier regression^12^) derived from a separate group of non-pregnant women recruited specifically for this purpose (the indicator group described in detail in^13^). To account for pregnancy-mediated changes in biomarkers the values were further adjusted for the gestational age of the infant at the time of measurement. This was achieved by regressing the biomarker on the first three orthogonal polynomials of gestational age and subtracting the predicted value so obtained from each woman’s seasonally adjusted value. Comparison of seasonal patterns in the plasma biomarker concentrations between the indicator and the main groups are shown in Fig. 1b and Supplementary Fig. 1.
2. Confirmation of the seasonal variation in biomarkers using analysis of variance: These data are shown in Supplementary Table 2.
3. Testing the validity of a single methylation score averaged overall six MEs (PARD6G excluded): This analysis was carried out using PBL DNA methylation data only, given the smaller HF data set. A simple score for the methylation percentage at each ME was derived for each infant by taking the logit of the mean methylation percentage overall CpG sites within the ME. We then fitted these simultaneously to each biomarker and covariated one at a time using seeming unrelated regression (SUR^20^). In each case we compared two models; one in which a different coefficient was fitted for each ME, and the other in which all the coefficients were constrained to be the same. In every case, except for offspring sex (SUR *P*=0.004), the unconstrained model fitted no better than the constrained one (that is, the biomarkers and other covariates did not have a differential effect on different MEs). Supplementary Table 4 shows the difference in individual ME methylation in males and females with generally lower methylation in males. These results justified the use of an overall methylation score for each infant, based on the sex-adjusted individual ME methylation scores, for subsequent analysis. The overall score was calculated by standardising each ME score (subtracting the sex-specific mean and dividing by the sex-specific standard deviation) and taking the mean overall six MEs.
4. Regression of MEs on maternal biomarkers and covariates: We used simple linear least squares regression to fit the methylation score to each biomarker, season and other covariates in turn. We also fitted two derived variables predicted *a priori* to be important in the regulation of methylation: SAM:SAH and BET:DMG ratios. These data are presented in Table 1. Two measures of effect size are presented to facilitate different interpretation: (i) the standardised β-coefficient, which gives the change in mean methylation score for each 1 s.d. change in the predictor and thus facilitates comparison of effect size between predictors and (ii) as the odds ratio, which was derived from the SUR analysis and gives the factor by which the odds of methylation, that is, percent methylation/(100-percent methylation), is expected to change for each unit change in the predictor. Significant maternal predictors of infant DNA methylation in PBL showed a consistent dose-responsiveness by maternal biomarker quartiles (Supplementary Fig. 3).
5. Exploring interactions between biomarkers: Simple models including the main effects for two variables and their interaction term were fitted (using PBL DNA methylation data). The motivation was to capture some of the complexities of one-carbon metabolism, for instance possible switching of the source of methyl groups between the betaine and the FOL-cycle. Since there is a prohibitively large number of biomarker pairs, we only examined a limited number of their interactions selected based on *a priori* knowledge of their relationships in the metabolic pathways: B2*FOL; B2*B12; B12*FOL; B2*HCY; B12*HCY; and FOL*HYC. Least angle regression, LASSO or other methods might have allowed us to select the best predictors among many correlated main effects and interactions. However, applying the LASSO to these data added little to the reported analysis (data not shown).
6. Estimation of the total association between biomarkers and methylation: Since no interactions between biomarkers or biomarkers and sex of the infant were found to be significant we calculated *R* ^2^ for the multiple regression of the methylation score on all the main biomarker terms. We then ran the same model on 1,000 bootstrap samples and used the 2.5th and 97.5th centiles of the *R* ^2^ estimates to derive the 95% confidence interval for *R* ^2^. Models with terms for season, sex of infant and age of mother yielded very similar partial *R* ^2^ values for the biomarkers so these terms were not included in the bootstrap analyses.
7. Testing for seasonal differences in biomarkers: To examine whether seasonal differences in methylation might be owing to seasonal difference in biomarkers we fitted a model including season and all biomarker main effects.

All biomarkers were analyzed in the logarithm. The main analysis was performed using Stata v12MEP_L_cop2 (StataCorp, College Station, TX, USA) and the sklearn package in Python was used to implement the LASSO.

## Author contributions

P.D.-S., S.E.M., S.E.C., A.J.F., R.A.W., A.M.P. and B.J.H. conceived and designed the study; P.D.-S., S.E.M. and B.J.H. conducted the research; P.D.-S. was involved in all hands-on experiments, and conducted the sample and data collection; P.D.-S., M.S., Y.G. and A.J.F. advised on or performed the statistical analyses; R.A.D., S.H.Z. and S.M.I. conducted the biochemical analyses; M.S.B., E.L. and R.A.W. carried out the epigenetic analyses; G.E.S. and A.W.B. provided the twin samples; P.D.-S., A.M.P., R.A.W., A.J.F. and B.J.H. drafted the manuscript and all authors critically revised and approved the manuscript.

## Additional information

**How to cite this article:** Dominguez-Salas, P. *et al.* Maternal nutrition at conception modulates DNA methylation of human metastable epialleles. *Nat. Commun.* 5:3746 doi: 10.1038/ncomms4746 (2014).

## Supplementary Material

Supplementary Information Supplementary Figures 1-4 and Supplementary Tables 1-4

## Figures and Tables

**Table 1:** Maternal predictors of mean methylation score across six metastable epialleles combined.

**Maternal Predictor**	**s.d.**	**PBL**					**HF**				
		**Stand β-coeff**	**95% CI**	**OR**	**95% CI**	***P*****-value**	**Stand β-coeff**	**95% CI**	**OR**	**95% CI**	***P*****-value**
BMI (kg m^−2^)	3.33	−0.12	−0.23 to −0.02	0.98	0.97–1.00	0.014*	−0.15	−0.29 to −0.02	0.98	0.96–1.00	0.024*
Age (years)	6.5	0.00	−0.10 to 0.10	1.00	0.99–1.01	0.968	−0.05	−0.19 to 0.09	1.00	0.99–1.01	0.472
FOL (nmol l^−1^)	0.39	0.02	−0.07 to 0.12	1.03	0.90–1.17	0.615	0.01	−0.11 to 0.13	1.00	0.86–1.16	0.813
B2 (1 EGRAC^−1^)	0.24	0.09	0.00 to 0.19	1.19	0.98–1.46	**0.046**	0.11	0.00 to 0.22	1.22	0.97–1.53	**0.042**
B12 (pmol l^−1^)	0.42	0.03	−0.07 to 0.14	1.04	0.91–1.19	0.539	0.08	−0.06 to 0.23	1.06	0.88–1.26	0.249
ACTB12 (pmol l^−1^)	0.49	−0.04	−0.16 to 0.07	0.98	0.87–1.11	0.454	−0.03	−0.18 to 0.13	1.00	0.85–1.18	0.749
CHOL (μmol l^−1^)	0.31	−0.01	−0.12 to 0.09	0.95	0.80–1.12	0.799	0.01	−0.13 to 0.14	0.96	0.77–1.19	0.909
BET (μmol l^−1^)	0.53	0.05	−0.10 to 0.20	1.03	0.89–1.19	0.485	0.13	−0.07 to 0.32	1.06	0.88–1.28	0.193
DMG (μmol l^−1^)	0.55	−0.06	−0.16 to 0.04	0.95	0.86–1.04	0.208	−0.02	−0.15 to 0.11	0.97	0.86–1.09	0.794
BET:DMG ratio	0.6	0.08	−0.02 to 0.17	1.05	0.97–1.14	0.113	0.06	−0.06 to 0.18	1.04	0.94–1.15	0.342
SAM (nmol l^−1^)	0.18	−0.06	−0.17 to 0.05	0.79	0.58–1.08	0.282	−0.05	−0.19 to 0.09	0.85	0.57–1.27	0.479
SAH (nmol l^−1^)	0.32	−0.09	−0.18 to 0.01	0.88	0.75–1.02	0.065	−0.12	−0.25 to 0.01	0.84	0.69–1.03	0.063
SAM:SAH ratio	0.3	0.06	−0.03 to 0.15	1.08	0.92–1.27	0.175	0.09	−0.03 to 0.22	1.15	0.93–1.41	0.132
MET (μmol l^−1^)	0.2	0.07	−0.03 to 0.18	1.19	0.90–1.56	0.178	0.00	−0.13 to 0.14	0.99	0.70–1.38	0.961
HCY (μmol l^−1^)	0.31	−0.14	−0.23 to −0.05	0.80	0.68–0.93	0.003**	−0.15	−0.27 to −0.03	0.82	0.67–1.00	0.015*
B6 (nmol l^−1^)	0.41	−0.16	−0.27 to −0.04	0.82	0.71–0.94	0.005**	−0.12	−0.26 to 0.02	0.86	0.73–1.02	0.080
CYS (μmol l^−1^)	0.15	−0.19	−0.31 to −0.07	0.45	0.30–0.68	0.002**	−0.20	−0.36 to −0.04	0.43	0.25–0.72	0.014*

ACTB12, active B12; BET, betaine; BMI, body mass index; CHOL, choline; CYS, cysteine; DMG, dimethyl glycine; EGRAC, erythrocyte glutathione reductase activity coefficient; FOL, folate; HF, hair follicle; HCY, homocysteine; MET, methionine; PBL, peripheral blood lymphocyte; SAH, S-adenosylhomocysteine; SAM, S-adenosylmethionine; s.d., standard deviation; Stand β-coef, standardized beta-coefficient.

The effect size is expressed as (i) standardized β-coefficient which describes the change in mean DNA methylation score per 1 s.d. of the predictor, as well as (ii) odds ratio (OR) which indicates the factor by which methylation changes for each unit change in the predictor (linear least squares regression models). Comparable and significant effects in PBL and HF DNA were obtained for B2, homocysteine and cysteine. Significant maternal predictors of infant DNA methylation in PBL showed a dose-responsiveness by maternal biomarker quartiles (Supplementary Fig. 3). *P*-values: *<0.05; **<0.01 are shown in bold; *N*_max_=126 for PBL data; *N*_max_=87 for HF data.
