# Supplementary material for: Maternal nutrition at conception modulates DNA methylation of human metastable epialleles
Source: Nat Commun. 2014 Apr 29;5:3746. doi: 10.1038/ncomms4746 (PMC4015319; doi:10.1038/ncomms4746)
Supplement: Supplementary Information — Supplementary Figures 1-4 and Supplementary Tables 1-4 [file ncomms4746-s1.pdf]

Supplementary Figure 1 Comparison of seasonal patterns in the concentrations of the remaining plasma biomarkers not shown in **Fig. 1b**.

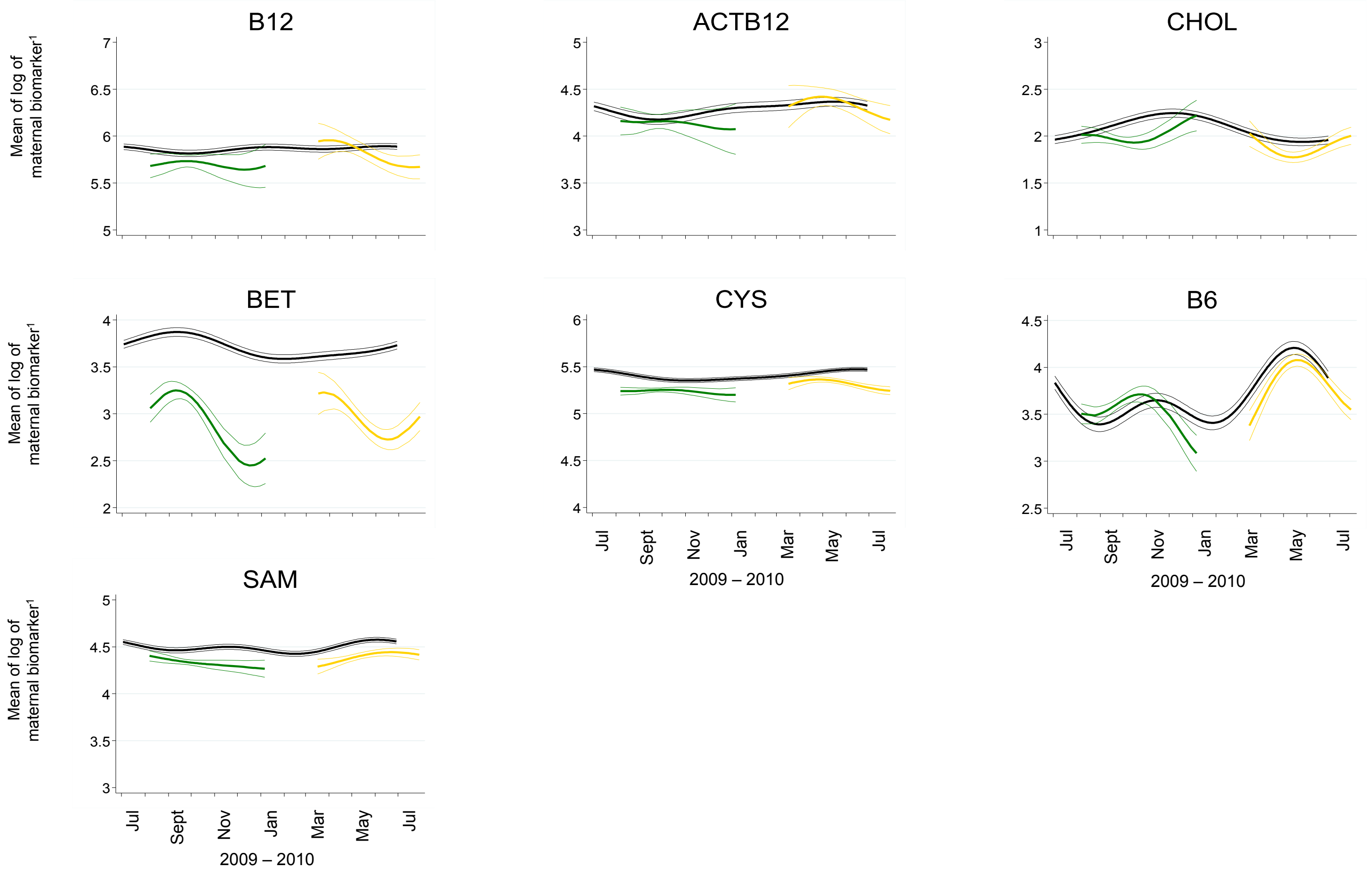

The graphs show the concentrations between the indicator group (N=30, shown in black) and the main group prior to back extrapolation (N=166 total; rainy season (green), dry season (yellow). Thick line, mean of logarithm of the biomarker; thin lines, 95%CI. [1] X-axis units: B6 and SAM in nmol l^{-1}; B12 and ACTB12 in pmol l^{-1}; BET, CHOL and CYS in µmol l^{-1}. ACTB12, active B12; BET, betaine; CHOL, choline; CYS, cysteine; SAM, S-adenosylmethionine.

Supplementary Figure 2 Systemic inter-individual variation in DNA methylation at human MEs.

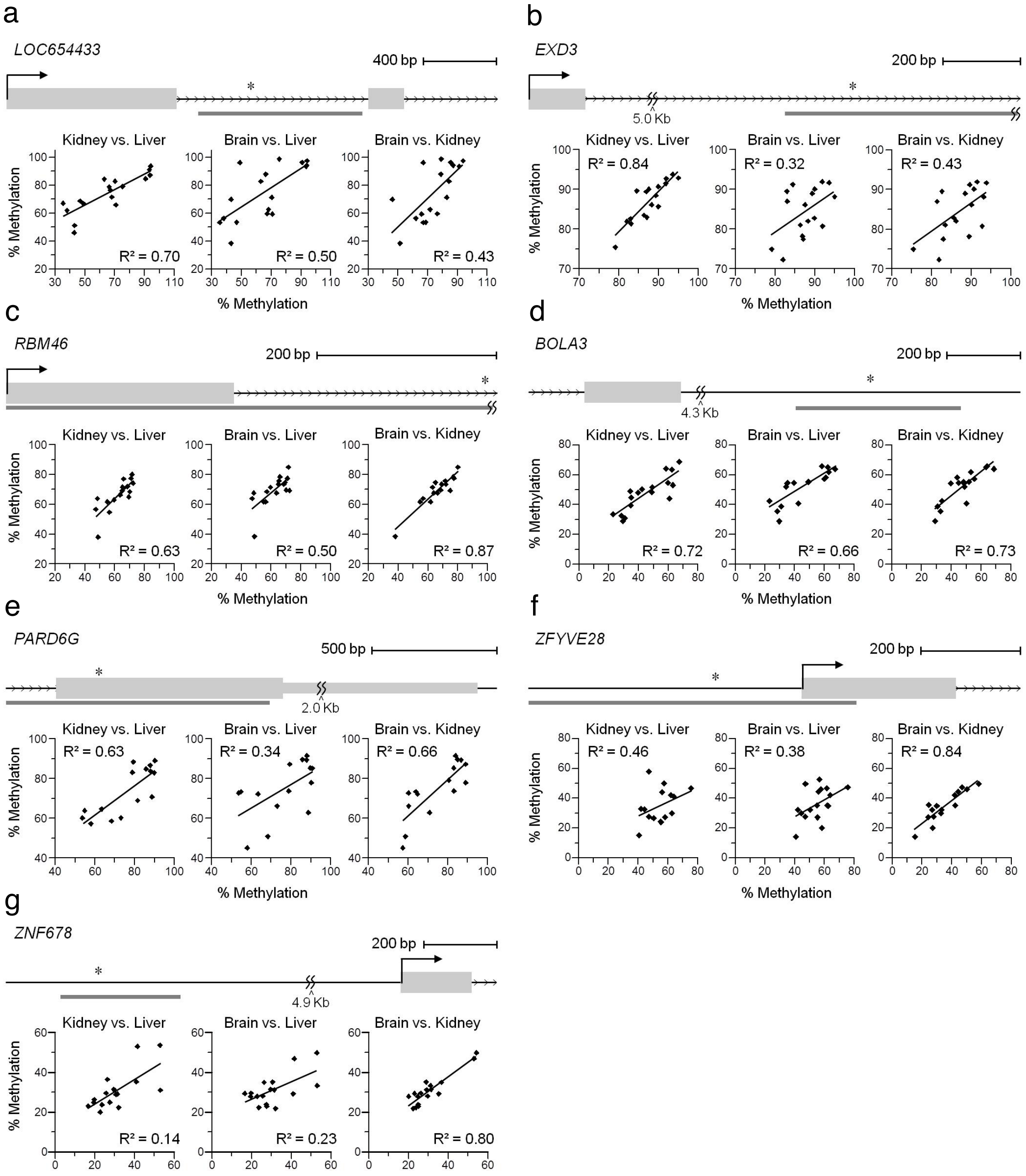

Scatter plots illustrating inter-tissue correlations among liver, kidney, and brain tissues of 17 Vietnamese at MEs (**a**) *LOC654433*, (**b**) *EXD3*, (**c**) *RBM46*, (**d**) *BOLA3*, (**e**) *PARD6G*, (**f**) *ZFYVE28*, and (**g**) *ZNF678*. The asterisk in the top of each panel indicates the specific location of the CpG sites assayed. Grey bars below the gene diagrams indicate CpG islands. At every locus, at least one of the three inter-tissue correlations yielded an R^2>0.50.

Supplementary Figure 3 Dose responsiveness of significant maternal predictors of infant DNA methylation in PBL (**Table 1**).

a

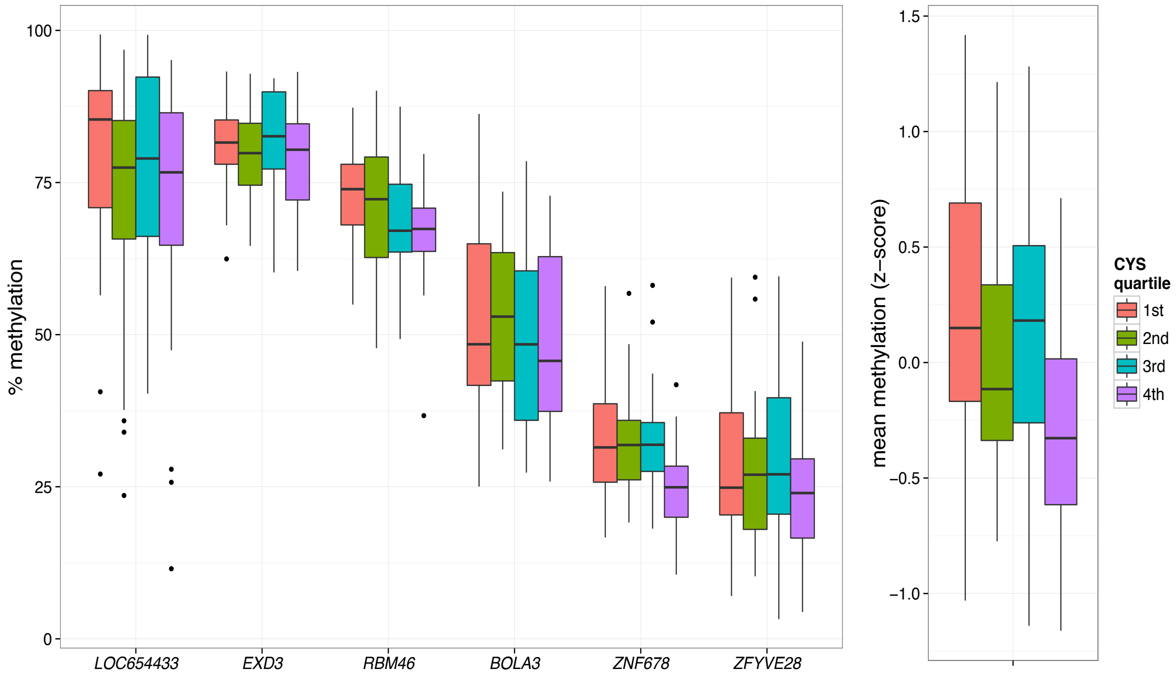

b

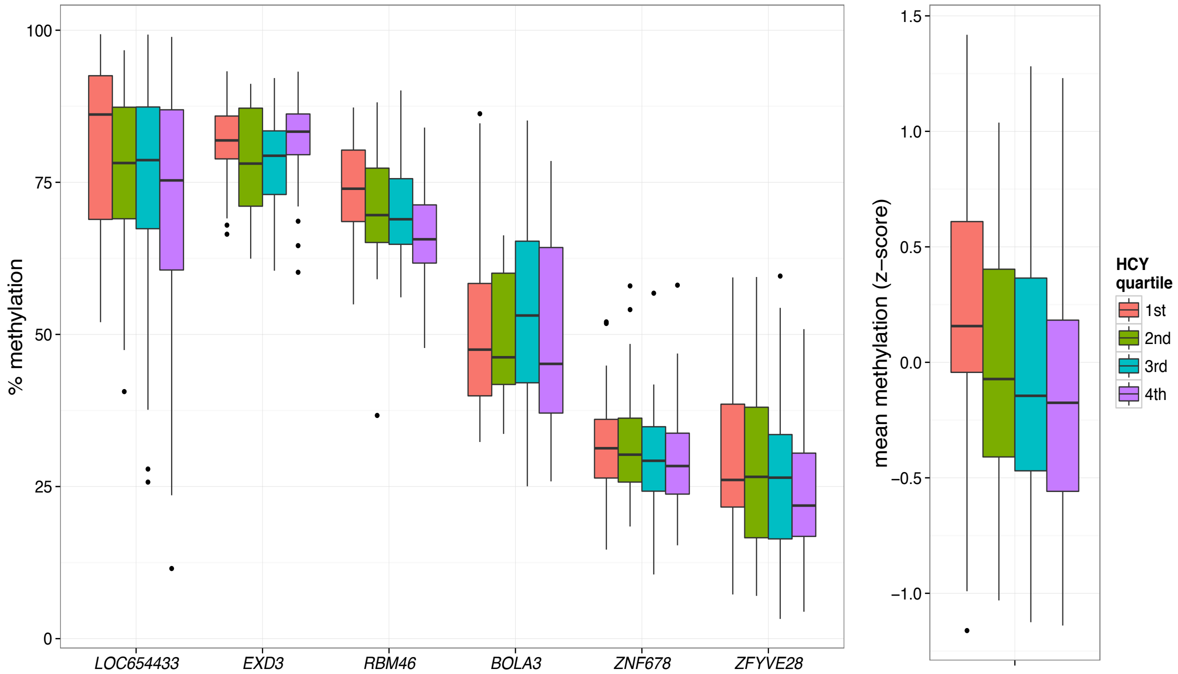

c

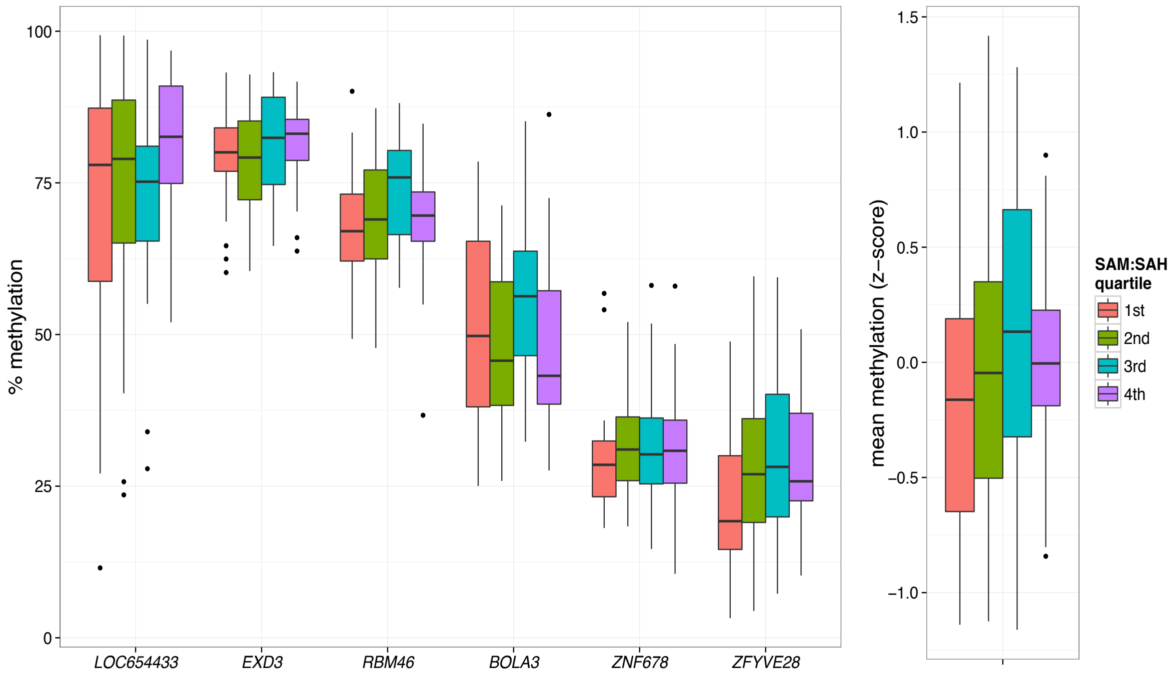

d

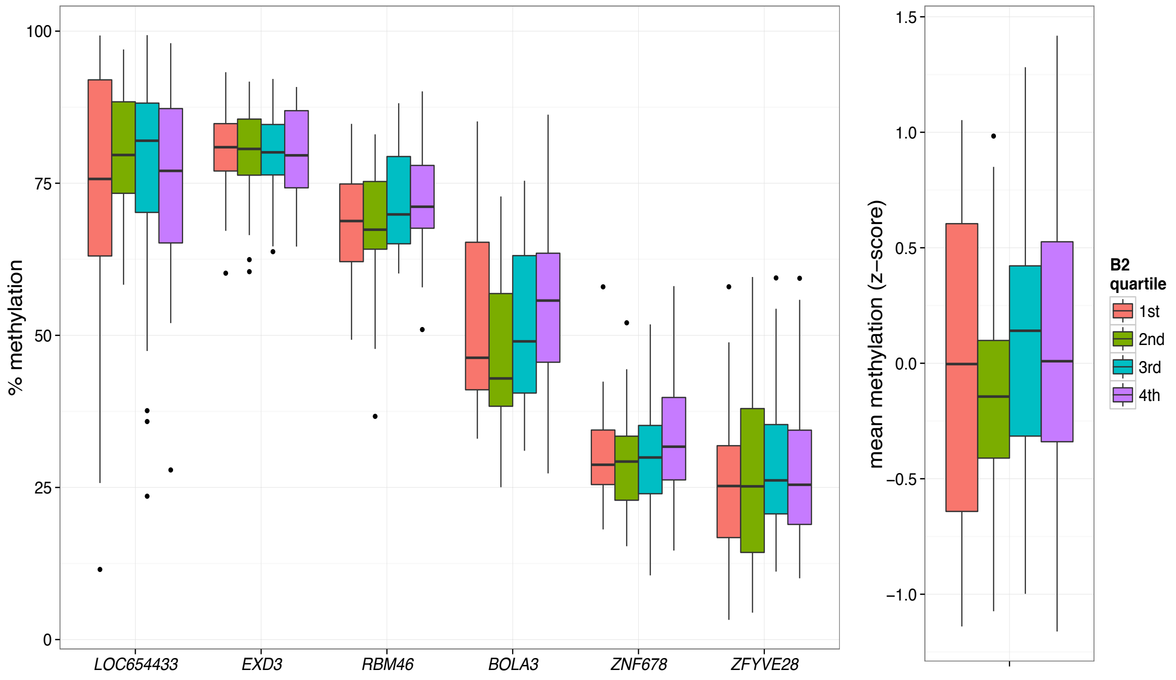

Box plots illustrate percent methylation at the six MEs by quartiles of maternal (**a**) Cysteine (CYS), (**b**) Homocysteine (HCY), (**c**) B6, and (**d**) B2. On the right of each panel is shown the mean methylation Z-score across the six MEs by quartiles of each maternal biomarker. Box plots represent the median (horizontal line) and interquartile range (box) of the indicated distribution. The whiskers extend from the top/bottom of the box to the highest/lowest data value that is within 1.5 * interquartile range of the box. Data beyond the whiskers are plotted as individual points. PBL, peripheral blood lymphocyte; N_{max}=126.

Supplementary Figure 4 Standard curves for the 7 ME pyrosequencing assays.

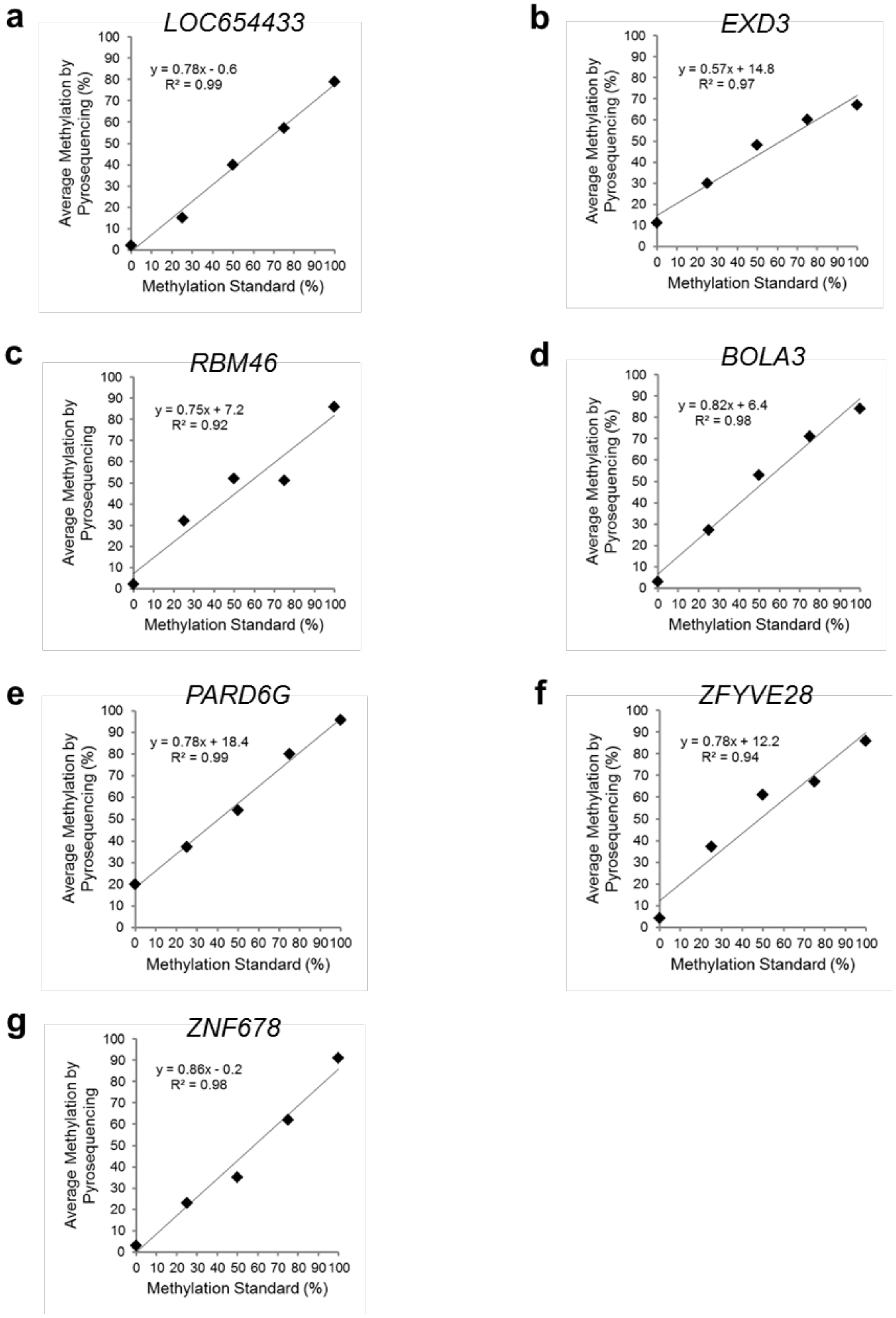

Supplementary Table 1 Demographic characteristics of the study population by season of conception

Characteristics	Rainy Season		Dry Season		P-value of difference between seasons
	N (%)	Mean (95% range)[b]	N (%)	Mean (95% range)[b]	
**Mothers**					
Age, years	83	29.3 (15.5-43.1)	83	29.1 (16.5-41.7)	0.838[c]
Ethnicity[a]					
Mandinka	70 (84.3%)	-	75 (90.4%)	-	
Fula	13 (15.7%)	-	7 (8.4%)	-	0.200[d]
Other	-	-	-	-	
Not known	-	-	1 (1.2)	-	
Sampling post conception, weeks	83	8.4 (-0.2-17.0)	83	8.9 (1.4-16.3)	0.434[c]
BMI at bleeding	83	21.2 (14.1-28.1)	83	21.1 (14.8-27.6)	0.789[c]
**Infants**					
Males	37 (55.2%)		35(51.5%)		0.662[d]
Age[e], months	64	3.8 (1.7-6.0)	57	3.1 (1.9-4.4)	<0.001[c***]
Ethnicity[a]					
Mandinka	46 (69.7%)	-	47 (78.3%)	-	
Fula	10 (15.2%)	-	5 (8.3%)	-	0.254[d]
Other	2 (3.0%)	-	-	-	
Not known	8 (12.1%)	-	8 (13.3%)	-	
Weight-for-length z-score	65	-0.5 (-3.4 - 2.5)	60	-0.4 (-2.5-1.8)	0.992[c]
Length-for-age z-score	65	-0.7 (-2.7-1.2)	58	-0.4 (-2.5-1.8)	0.054[c*]

A total of 166 mothers participated in the study. A total of 126 of their infants were recruited at 2-9 months (3.6 ± 1.2) [mean ± SD] of age. Loss to follow-up in the infant group was due to miscarriage, death, withdrawal by their parent or guardian, or difficulty to collect biological sample(s). Data missingness in the maternal or infant groups was due to failure to collect baseline information in a few individuals. The majority of characteristics between the rainy and dry season mother and infant groups were not significantly different. Infants conceived in the rainy season were visited slightly later than those conceived in the dry season and had marginally greater length-for-age z-scores. BMI, body mass index; [a] (self-)reported, based on father's ethnicity; [b] 95% range = Mean ± 2SD; [c] Wilcoxon-Mann-Whitney non-parametric test; [d] Chi-square test; P-values: * <0.05; ** <0.01, ** <0.001; [e] at sampling

Supplementary Table 2 Differences in plasma biomarker concentration between women conceiving during the rainy versus dry season, with adjustment for time of bleeding (approximation to conception time) and effect of week of gestation

Biomarker	N	GM[a] in rainy season[b] (95%CI)	GM[a] in dry season[b] (95%CI)	Between season difference
**Folate** [nmol l^{-1}]	163	18.60 (17.02-20.33)	12.91 (11.96-13.94)	**5.69*****
**B2 deficiency** [1 $EGRAC^{-1}$]	155	0.51 (0.48-0.54)	0.41 (0.39-0.43)	**0.10*****
**B12** [pmol l^{-1}]	164	306.70 (279.97-335.98)	335.49 (308.80-264.47)	-28.79
**Active B12** [pmol l^{-1}]	164	64.29 (57.76-56.27)	77.15 (69.92-85.13)	**-12.86***
**Choline** [μmol l^{-1}]	163	6.54 (6.17-6.94)	6.88 (6.42-7.38)	-0.34
**Betaine** [μmol l^{-1}]	164	20.64 (19.35-22.02)	17.53 (16.02-19.18)	**3.11****
**DMG** [μmol l^{-1}]	164	1.70 (1.52-1.91)	2.88 (2.58-3.22)	**-1.18*****
**BET:DMG**	164	12.22 (10.84-13.77)	6.06 (5.38-6.82)	**6.16*****
**Methionine** [μmol l^{-1}]	164	25.43 (24.35-26.56)	22.66 (21.78-23.57)	**2.77*****
**SAM** [nmol l^{-1}]	163	76.99 (74.10-79.99)	75.89 (73.05-78.84)	1.10
**SAH** [nmol l^{-1}]	163	8.59 (8.12-9.09)	11.67 (10.89-12.52)	**-3.08*****
**SAM:SAH**	163	8.96 (8.45-9.50)	6.52 (6.12-6.95)	**2.43*****
**Homocysteine** [μmol l^{-1}]	163	6.11 (5.73-6.52)	7.67 (7.17-8.22)	**-1.56*****
**B6** [nmol l^{-1}]	166	33.82 (31.80-35.97)	36.18 (32.58-40.18)	-2.36
**Cysteine** [μmol l^{-1}]	164	198.82 (193.35-204.44)	197.24 (192.12-202.50)	1.58

DMG and SAH were also adjusted for time to processing (time between blood collection and plasma storage at -80°C). Biomarkers patterns with a P<0.001 (ANOVA) for seasonal difference are illustrated in **Fig. 1b**, the remaining biomarkers are shown in **Fig. S1**. [a] Geometric mean; [b] Rainy season: July-September 2009; Dry season: February-April 2010; ANOVA P-values: * <0.05; ** <0.01, *** <0.001; EGRAC, erythrocyte glutathione reductase activity coefficient; DMG, dimethylglycine; BET:DMG, betaine:DMG ratio; Hcy, homocysteine; SAH, S-adenosylhomocysteine; SAM, S-adenosylmethionine.

Supplementary Table 3 Details of the PCR and pyrosequencing conditions for DNA methylation analyses

Locus ID [a] (chromosomal position)	N of CpG sites	Forward Primer	Reverse Primer	Product size [bp]	Annealing temperature [°C]	Pyrosequencing Primer
BOLA3 (5kb downstream) chr2:74,357,770	5	GGTGTATTTAAGTATAGAG AAGGTGGAGAT	Btn-AAAACAACTAAAACTCAC AAACCACTACTA	206	61	GTGGAGATGGAGGGA
LOC654433 (Intron 9) chr2:113,992,770	5	GGGGTGGATGAGATTGAG GTTAGA	Btn-CCTTCAATACCTTTCCCC ATACTACC	171	67	GGTTTGTTTTGAGGAT
RBM46 (Intron 1) chr4:155,703,000	12	TTGTATGGTGAGGGTTTAG	Btn-TCTAAAACCAAACTACTAA ATCT	293	51	GTGTTATTTTTTTGATA
ZFYVE28 (Promoter CGI) chr4:2,366,687	5	TTTAGTAGGGGYGGYGTAG TTTTAGTTATA	Btn-AAACCTAACRCCTAAAAA ATAACC	82	59	GGYGTAGTTTTAGTTATAGA GT
EXD3 (Intron 1) chr9:140,312,195	5	Btn-AGGTTATTGAGTTGGGTTT TTT	CTCCTCAAATCCTCAAAC TCTATCC	134	53	CCTCAAACTCTATCCTTTC
PARD6G (Last exon) chr18:77,918,167	6	GTAGATGGAATAGTAGTGT TAGGTGTATGA	Btn-TCATTCACAACCAACAAC C	437	53	GGGTTGGAGAAGGTG
ZNF678 (5kb upstream) chr1:227,746,190	4	Btn-GGGTGTTGAAGGTTTTTT	CAAACCAAACTCCTAATA CTAT	220	55	CTACCTAATAATACTACAATC

[a] Based on Human Genome Build 19; Btn, 5' biotinylated; Chr, chromosome; CGI, CpG island; CpG, cytosine-phosphate-guanine, all primers listed 5' to 3'

Supplementary Table 4 Sex differences in methylation at six metastable epialleles.

Locus ID	OR	95%CI
BOLA3	0.802	0.653 - 0.987
LOC654433	0.901	0.611 - 1.329
RBM46	0.807	0.693 - 0.940
ZFYVE28	1.123	0.883 - 1.428
EXD3	1.233	1.040 - 1.462
ZNF678	0.802	0.694 - 0.926

Differences in ME methylation (SUR regression) in males and females are shown as OR, with lower methylation in males for four out of the six MEs. N_{max}=126 PBL DNA samples
